# *Assessing outcomes of enhanced chronic disease care through patient education and a value-based formulary* study (ACCESS)—study protocol for a 2×2 factorial randomized trial

**DOI:** 10.1186/s13012-016-0491-6

**Published:** 2016-09-26

**Authors:** David J. T. Campbell, Marcello Tonelli, Brenda Hemmelgarn, Chad Mitchell, Ross Tsuyuki, Noah Ivers, Tavis Campbell, Raj Pannu, Eric Verkerke, Scott Klarenbach, Kathryn King-Shier, Peter Faris, Derek Exner, Vikas Chaubey, Braden Manns, On behalf of the Interdisciplinary Chronic Disease Collaboration

**Affiliations:** 1 Departments of Medicine and Community Health Sciences, Cumming School of Medicine, University of Calgary, Calgary, Canada; 2 Departments of Medicine and Community Health Sciences, Libin Institute and Institute for Public Health, Cumming School of Medicine, University of Calgary, Calgary, Canada; 3 Alberta Health, Edmonton, Canada; 4 Department of Pharmaceutical Sciences, University of Alberta, Edmonton, Canada; 5 Department of Family Medicine, University of Toronto, Toronto, Canada; 6 Department of Psychology, University of Calgary, Calgary, Canada; 7 Emergence Creative, New york, USA; 8 Department of Medicine, University of Alberta, Edmonton, Canada; 9 Faculty of Nursing, University of Calgary, Calgary, Canada; 10 Alberta Health Services, Edmonton, Canada; 11 Department of Medicine, University of British Columbia, Vancouver, Canada; 12 Foothills Medical Centre, 1403 29th St. NW, Calgary, Alberta Canada T2N 2T9

**Keywords:** Randomized clinical trial, Chronic disease, Cost, Education, Drug insurance

## Abstract

**Background:**

Chronic diseases result in significant morbidity and costs. Although medications and lifestyle changes are effective for improving outcomes in chronic diseases, many patients do not receive these treatments, in part because of financial barriers, patient and provider-level knowledge gaps, and low patient motivation. The *Assessing outcomes of enhanced chronic disease care through patient education and a value-based formulary* study (ACCESS) will determine the impact of two interventions: (1) a value-based formulary which eliminates copayment for high-value preventive medications; and (2) a comprehensive self-management support program aimed at promoting health behavior change and medication adherence, combined with relay of information on medication use to healthcare providers, on cardiovascular events and/or mortality in low-income seniors with elevated cardiovascular risk.

**Methods:**

The ACCESS study will use a parallel, open label, factorial randomized trial design, with blinded endpoint evaluation in 4714 participants who are over age >65 (and therefore have drug insurance provided by Alberta Blue Cross with 30 % co-payment); are at a high risk for cardiovascular events based on a history of any one of the following: coronary heart disease, prior stroke, chronic kidney disease, heart failure, or any two of the following: current cigarette smoking, diabetes mellitus, hypertension, or hypercholesterolemia; and have a household income <Can$50,000. This 3-year study is powered to detect a minimal clinically important relative risk reduction of 12 % in the composite clinical outcome of all-cause mortality, nonfatal myocardial infarction, nonfatal stroke, need for coronary revascularization, and hospitalizations for chronic disease-related ambulatory care sensitive conditions, each of which will be assessed using healthcare administrative data. Secondary outcomes will include quality of life and healthcare costs.

**Discussion:**

Given identified gaps in care in chronic disease, and the frequency of financial and knowledge-related barriers in low-income Albertans, this study will test the impact of providing free high-value preventive medications (i.e., value-based insurance) and a tailored self-management education and facilitated relay strategy on outcomes and costs. By measuring the impact on both health outcomes and costs, as well as the impact on reducing health inequities in this vulnerable population, our study will facilitate informed policy decisions.

**Trial registration:**

Clinicaltrials.gov: NCT02579655. Registered Oct 15, 2015.

**Electronic supplementary material:**

The online version of this article (doi:10.1186/s13012-016-0491-6) contains supplementary material, which is available to authorized users.

## Background

The World Health Organization estimates that nearly one third of worldwide deaths are from cardiovascular disease [1]. Approximately 80 % of these are from myocardial infarctions and strokes. Similar statistics have been demonstrated in Canada [2]. Important risk factors for cardiovascular events include prior stroke or myocardial infarction, chronic kidney disease, heart failure, diabetes, or hypertension (henceforth referred to as “chronic diseases”).

Health behavior changes (e.g., exercise, healthy diet, smoking cessation, and adherence with pharmacologic preventive management) are particularly important in the management of people with these chronic diseases [3–7]. We identified a significant evidence to care gap for people with chronic diseases in Alberta, Canada, similar to that observed in other North American populations, including that nearly 50 % of people with cardiovascular-related chronic diseases do not take guideline-recommended medications (e.g., statins) [8].

As occurs in nearly all organizations for economic co-operation and development countries [9], most Canadian citizens (irrespective of whether they have private or publicly funded drug insurance) are subject to cost-sharing (i.e., copayments or deductibles) [10]. This requires that patients pay a portion of the medication cost—typically a copayment of 20–30 % of the total cost of the prescription [11]—or deductibles which may be as high as 5–20 % of household income [12]. In a recent survey of nearly 2000 Western Canadians, up to 20 % of Western Canadian patients identified having financial barriers to obtaining their drugs; and patients with these barriers were 30–55 % less likely to use statins than those who did not perceive financial barriers [10].

In addition to financial barriers, in our recent survey of Western Canadians, we also identified that patient and provider-level knowledge gaps and patient motivation were major barriers to optimal medical management [13]. Patient knowledge and motivation both tie into the concept of self-management [14]: a set of positive health behaviors which include adherence to prescribed medications. Our recent survey suggested that 40 % of patients with these chronic conditions did not recall receiving health behavior change counseling, and of those who did, up to 70 % of them did not report following the advice given [13].

Management of chronic disease may be suboptimal when patients experience financial barriers or do not engage in self-management. If suboptimal control persists for extended periods, patients may be hospitalized for complications [15] such as myocardial infarction, stroke, coronary revascularization, or chronic disease-related ambulatory care sensitive conditions (ACSC). ACSC are those for which “timely and effective outpatient care, including use of appropriate medications, can reduce the risk of hospitalization” [16]. These potentially preventable hospitalizations are clinically relevant to patients and for health systems [17–19].

### Rationale for study interventions

- Value-based formulary which eliminates copayment for high-value preventive medications

One way to reduce the frequency of financial barriers is to provide full coverage for “high-value” medications. Also known as value-based insurance, within a value-based formulary, medications shown to confer important benefits in high quality studies, and/or provide good value for money are provided for free, with other lower-value medications still subject to patient copayment [20]. Numerous observational studies have suggested that reducing copayments has the potential to improve adherence [20], and a recent high-quality randomized clinical trial (MI-FREEE) has demonstrated that eliminating copayments for beta-blockers, angiotensin converting enzyme inhibitors, statins, and antiplatelet agents in patients post-myocardial infarction reduced the total number of cardiovascular events [21]. The impact of value-based insurance in a broader patient population has not been tested using a rigorous study design. The ACCESS study is powered to determine the impact of value based insurance in a broader patient population on both clinical outcomes and costs—and as such will address an important gap in the literature.

b) Self-management support program

A variety of interventions to enhance patient self-management have been tested in people with chronic diseases within randomized clinical trials [22]. Within a recent systematic review that sought to determine which elements of such programs were most effective in people with diabetes, interventions to enhance self-management, patient education, and facilitated relay of information to clinicians were noted to be some of the most effective interventions at improving glycemic control and blood pressure [22]. “Facilitated relay” is defined as clinical information transmitted to clinicians by means other than the existing medical record [22]; the expectation is that clinicians act on the information to improve patient management. The systematic review noted considerable heterogeneity in the observed efficacy of the self-management programs. Part of this difference may relate to the format of the message, which (when crafted and delivered by health care workers) may lack effective techniques demonstrated to promote behavior change, and may not be tailored appropriately to individuals’ preferences and values [23]. Additional uncertainty with respect to the effectiveness of interventions to enhance self-management, patient education, and facilitated relay of information to clinicians was also noted because of low sample sizes and short study duration (almost all studies <6 months) [22]. This uncertainty will be addressed by the ACCESS study, which will be powered to detect differences in clinical and cost outcomes.

### Objectives

The *Assessing outcomes of enhanced chronic disease care through patient education and a value-based formulary* study (ACCESS) will determine the effectiveness of two interventions targeting financial and self-management related barriers:

(i) a value-based formulary that eliminates co-payment for select high-value medications (i.e., value based insurance); and
(ii) a comprehensive self-management support program aimed at promoting health behavior change (by addressing concerns and improving self-efficacy), combined with relay of information on medication use to health care providers on cardiovascular events and/or mortality in low-income seniors with elevated cardiovascular risk.

### Trial design

ACCESS is a pragmatic, parallel group, open-label, factorial randomized controlled trial with blinded endpoint evaluation of the two interventions described above. In this 2×2 design, there will be four treatment arms with an allocation ratio of 1:1:1:1. We will assess the impact of these interventions on both relevant clinical outcomes and healthcare system costs over a 3-year follow-up period.

## Methods

### Study setting

The ACCESS trial will recruit community-based participants living in Alberta, the fourth largest Canadian province with a population of 4.2 million [24]. In Canada, universal public health insurance provides physician and hospital services free of charge to all citizens and residents, but Alberta government-sponsored drug insurance is only provided without premium to people receiving social assistance and those over the age of 65 years. Those over age 65 pay 30 % copayment for drugs (to a maximum of $25 per prescription).

### Eligibility criteria

Inclusion criteria include: (a) age greater than 65 years with Alberta government-sponsored seniors drug insurance (30 % copayment), (b) high cardiovascular risk based on a history of any one of: heart disease, stroke, chronic kidney disease, heart failure, or any two of: current smoking, diabetes, hypertension, or high cholesterol; and (c) household income <$50,000.

Exclusion criteria include: (a) coverage by a secondary insurance plan (in addition to Blue Cross), resulting in patient-borne copayment of <30 %, or (b) inability to participate in self-management modules due to cognitive impairment or a lack of an English-speaking family member or close friend.

### Goals of interventions

The overarching purposes of the interventions in the ACCESS study will be to:

1. Increase initiation and adherence to medications that have been proven to reduce the risk of cardiovascular events in this population of high risk patients, including HMG-CoA reductase inhibitors (Statins) [25] and renin-angiotensin-aldosterone system inhibitors (ACE inhibitors [ACEi] and angiotensin receptor blockers [ARBs]) [26].
2. Encourage participants to make positive health behavior changes, including healthy dietary choices, engagement in physical activity, cessation of tobacco use, and increased adherence to all preventive medications.

*Intervention 1* will be enrolment in a new drug formulary (operationalized through their existing government drug insurance) that will eliminate copayments for high value preventive medications (those which prevent myocardial infarction, strokes, hospitalizations and delay progression of kidney and other vascular disease): statins, beta blockers, ACE-i, ARBs, calcium channel blockers, diuretics, anti-platelet agents, anti-arrhythmic drugs, anticoagulants, oral anti-diabetes agents, insulins, and smoking cessation aids (Additional file 1: Appendix 1).

*Intervention 2* will be a tailored, adaptive, and interactive self-management support system that attempts to address determinants of intention and self-efficacy with regards to improving health habits and medication taking behavior. The second component of this intervention is comprised of facilitated relay of participants’ current use of recommended medications to their regular healthcare providers to facilitate prescription changes as needed, with the goal of overcoming clinical inertia.

The system was designed by behavioral and implementation scientists, clinical experts, health services researchers, in partnership with a health marketing firm (Emergence, New York, NY) and a health technology firm (Locus Health, Charlottesville, VA) to incorporate principles of design-thinking and brand-engagement, informed by a process of patient engagement, as described below. Details, including the final design and implementation plan, are presented in the Box.

### Box: Intervention 2 design and implementation

Step 1—defining the objective: the primary objective of intervention 2 was to improve patient knowledge and participants’ self-management abilities including making positive health behavior changes including adherence to prescribed medications (especially statins and ACE-inhibitors/ARBs), healthy lifestyle choices, and smoking cessation for those who self-report smoking cigarettes.

Step 2—initial patient engagement: we conducted a series of ethnographic-style interviews where one academic researcher (DC, BM, TC) was paired with a marketing expert to visit the homes of ten purposively sampled community dwelling patients chosen on the basis of having low incomes and several chronic conditions that would qualify them for inclusion in this trial. Questions focused on their medical conditions and medications, to explore the perceived meaning and impacts of these in their daily lives. From this experience, we developed different patient phenotypes or archetypes, which was foundational to developing the intervention [27].

Step 3—tailoring the intervention: beyond these patient archetypes, we used several individual measures to tailor the intervention for individuals’ situation. (1) The Necessity-Concerns framework [28] was used to personalize the intervention to individuals’ circumstances and beliefs. We stratified individuals into categories of necessity-predominant beliefs (those who don’t believe their medications are necessary or helpful) and concerns-predominant beliefs (those who have concerns about side-effects, dependency or interactions). (2) Given that different types of messaging might be required for those who are non-adherent to their medications, we used validated instruments for assessing adherence at study baseline (Morisky Adherence Questionnaire) [29] to assist in categorizing participants appropriately.

Step 4—procuring patient recommendations: we searched publicly accessible websites from professional societies and patient-information groups to obtain the medical information appropriate for trial participants. These included dietary and physical activity recommendations, scientifically-sound explanations of medication effects, and evidence of medication efficacy. These materials were reviewed by clinical committee members for appropriateness for inclusion.

Step 5—brand and message development: based on insights generated from the immersive patient research, the marketing team created a patient-facing brand and visual identity for the engagement platform. They then transformed physician-vetted medical information into messages that are easily understandable and relatable by the majority of potential participants. Specific messaging was developed to address each different patient archetype, adherence-state and beliefs-state described above.

Step 6—development of the online platform: the marketing team in conjunction with Locus Health, the health technology partner, then designed, developed and built an online portal for patients to interface with the design elements and messaging material developed in step 5. Participants who indicate a preference for an electronic mode of communication receive an email on a daily basis inviting them into their portal where they receive information, or are asked to respond to as series of cards. For each card completed, participants receive points which are tracked and make them eligible to receive virtual awards. This approach is designed to improve engagement with the platform through positive reinforcement.

Step 7—development of the analog platform: from previous studies in the area, we expected that at least half of study participants would not be able or willing to access the online platform [30]. From the types of messaging developed in step 5, our marketing partners developed paper-based mailers that participants randomized to receive the self-management intervention receive weekly focusing on medication adherence and positive health behavior choices.

Step 8—development of the facilitated relay strategy: since only 50 % of Albertans with chronic diseases are taking guideline-recommended therapies [10, 31], we developed personalized letters that would be sent to participants to take to their primary care provider and to their pharmacist. Where individuals are not currently prescribed both classes of recommended medications, the purpose of the letter is to remind the care provider about the evidence behind these therapies and strategies to attempt if the drugs had previously not been tolerated. For those who are on both statins and ACEi/ARBs at baseline, the letter simply informs their provider that they are enrolled in a clinical trial with a self-management intervention designed to assist them in working with the patient for improved heart health. These letters were pilot-tested with five primary care physicians and four pharmacists and refined based on the feedback received.

Step 9—focus group testing: once a preliminary version of the intervention was designed, we convened two focus groups of individuals (*n* = 6 and *n* = 8) who would meet inclusion criteria for the trial. We solicited their feedback about the various elements of the intervention (including the brand identity and visual design), receiving important comments and suggestions. For example, we heard that our initial design was too complex and that navigating the site was not intuitive. We also heard that the brand name of the platform was unpalatable and not easily relatable to focus group participants.

Step 10—intervention redesign: based on the feedback received in step 9, the marketing and health technology teams made further changes to the delivery platform. In response to feedback about overly complex design, the intervention was simplified and streamlined to increase usability. The platform was also renamed and rebranded as a result of focus group feedback.

Step 11—beta testing: 20 individuals were recruited to beta test the platform, including members of the study team, as well as older adults with chronic diseases. Users were given access to the electronic platform for a two-week period at which point all users were asked to complete a debriefing questionnaire, and qualitative debriefing interviews were undertaken with participating seniors.

Step 12—finalization of the components and implementation strategy for intervention 2: after completion of the above steps, we finalized the self-management intervention and describe below an overview of the patient experience:

- Week 1—participants receive a letter informing them that they have been randomized to receive the personalized self-management intervention.
- Week 2—participants receive a designed, personalized starter kit welcoming them to the informational platform, including:
  - Information guide booklet with information on how to log in to the online platform
  - Quick start guide
  - Pedometer
  - Wallet card to record user name and password
  - Facilitated relay letters to take to primary care physician and pharmacist, tailored based on whether the patient is receiving statins and/or ACEi/ARB at baseline, and if not, whether they have tried them previously and experienced side-effects or had significant medication-related concerns as identified on their responses to the Beliefs about Medications Questionnaire. For patients not receiving either statins or ACEi/ARB, the initial letter focuses on statin use, with a subsequent letter about ACEi/ARB use sent at 3 months.

Daily throughout study—participants receive an email with personalized messages around medication, adherence, relevant health behaviors or their specific chronic conditions. These emails encourage them to log in to their secure online portal for further information.

NOTE: those who choose to receive their intervention through non-electronic means receive weekly mailers instead of daily emails.

At 1 month—participants receive a letter reminding them to take their initial facilitated relay letters to their physician and pharmacist.

Monthly—all participants receive paper-based mailers through the postal mail system. These are designed to drive engagement with the platform and contain general information about healthy living, including anecdotes, recipes, and suggestions.

At 3 months—to decrease the risk of disengagement, participants receive a free gift (a branded re-useable shopping bag) thanking them for participating and encouraging them to continue.

At 3 months—participants who were not on either statins or ACEi/ARBs at baseline receive their second set of facilitated relay letters (on the importance of ACEi/ARB use) to take to their healthcare providers.

At 6 months—participants receive a menu in the mail inviting them to select one of two free gifts, which include a new upgraded pedometer, or a tailored and branded health tracking book.

At 6 months—participants also complete their 6-month follow-up survey, including updated information on medication use and adherence—which will inform future interactions with the platform.

Daily/weekly/monthly interactions continue through the duration of the trial.

### Control arms

The control arm for intervention 1 (copayment elimination) is the usual pharmaceutical insurance plan. Those who are randomized to usual insurance coverage continue to have access to their regularly prescribed medications through their Alberta Blue Cross seniors plan with their usual 30 % copayment to a maximum of $25 per prescription.

The control for intervention 2 (the self-management intervention) has access to generic chronic disease information. Participants in this study arm are provided with a link to a government website (myhealth.alberta.ca) housing health information on a variety of conditions written by the company Healthwise. Participants can find information that interests them on a self-directed basis. They are also automatically subscribed to receive the quarterly patient-focused magazine published by Alberta Health Services.

### Outcomes

The primary outcome is the composite rate of all-cause mortality, MI, stroke, coronary revascularization (coronary artery bypass grafting, angioplasty, or coronary stenting), and hospitalizations for cardiovascular-related ambulatory care-sensitive conditions defined using validated algorithms through administrative data [16] (Additional file 1: Appendix 2). As an individual may experience multiple events, a rate outcome will be used rather than a binary composite. Secondary outcomes include: (1) individual components of the primary endpoint, (2) changes to medication adherence, medication self-efficacy and needs-concerns states (see below), and (3) overall quality of life. Alongside the clinical trial, we are also planning a cost-effectiveness analysis, and a qualitative descriptive study, both described briefly in separate sections below.

### Sample size

We identified a cohort of 170,000 Alberta seniors from the Interdisciplinary Chronic Disease Collaboration (ICDC) Chronic Disease Repository (Additional file 1: Appendix 3) who would meet study entry criteria. We found that the annual composite primary outcome event rate was estimated as 14 per 100 participant years. We used this rate to determine sample size requirements [32], assuming a minimal clinically important relative risk reduction of 12, *α* = 5 %, 80 % power, allocation ratio of 1:1, average follow up of 3 years and presumed 1 percent per year loss to follow-up (due to outmigration from Alberta) [33]. Also, 4714 patients are required, assuming no important interaction between our two interventions, which was tested using simulations with plausible interactions of 25 and 50 %.

### Recruitment strategies

Participants will be recruited via signage in primary care and specialist physician offices as well as in pharmacies. Several hundred pharmacies across the province have agreed to participate in recruitment for this trial by posting study posters and by handing recruitment brochures to any patients filling medications that would suggest they might be eligible for this study (anti-hypertensive or anti-diabetes medications). We will also recruit using local newspapers and community newsletters, through senior’s groups as well as mass media (television, radio and social media).

### Sequence generation and allocation concealment

Once baseline surveys have been returned to the study team and we have verified eligibility, randomization to the interventions will occur in a 1:1:1:1 fashion via a computer generated randomization program.

A randomized treatment allocation table was created on October 28, 2015 using sealedenvelope.com. Four treatment groups were used (intervention 1 only, intervention 2 only, interventions 1 and 2, and no intervention/control). Variable block sizes were used and the list length was 7200. Randomization was stratified by age (<70 years, ≥70 years); income (< $30,000, ≥$30,000); and sex (Male, Female).

Study data will be collected and managed using REDCap electronic data capture tools hosted at the University of Calgary, in collaboration with the Cumming School of Medicine. The randomization module was enabled in REDCap, and the allocation table produced by Sealed Envelope was imported into the web-based application. Patients will be entered into the database and randomized, in a concealed fashion, according to this table by the study coordinating centre.

### Blinding

Given the pragmatic nature of copayment elimination, blinding is not possible for this intervention. For the self-management intervention, even control patients will receive a basic version of potentially relevant educational materials, in essence being single-blinded for this part of the study.

However, to deal with the risk of ascertainment bias in this observational study, administrative data codes defining the primary and secondary outcomes were defined in advance (Additional file 1: Appendix 2) and outcome assessment will be blinded by having an analyst who does not have access to treatment information.

### Data collection

Subjective outcomes such as quality of life, self-efficacy, and needs-concerns will be obtained from surveys administered at baseline, 6, 18, and 36 months (study completion). Objective outcomes will be assessed using the ICDC Chronic Disease Repository [32], which includes provincial laboratory and administrative health data (including vital statistics, pharmacy claims, physician claims, hospitalizations, emergency department and outpatient visits, and all health care costs) for all Albertans (Additional file 1: Appendix 3). This dataset has been used for many observational studies [34–37] and for assessing outcomes in a RCT of over 20,000 individuals [33].

Primary endpoint: the use of administrative databases to identify clinical conditions and outcomes has been validated in numerous high-quality studies [38–40], and the specific ICD-10 codes we have chosen to define the outcomes have been endorsed by respected national organizations [41] and based on validated algorithms applied to administrative health data [38, 40, 42–47].

Secondary endpoints:

1. Medication adherence: measured by the “number of days dispensed”/“number of days between prescription renewals” [48, 49] using drug data in the ICDC Chronic Disease Repository. Patients with medication supplies to cover ≥80 % of observed treatment days are considered adherent [21, 50].
2. Quality of life: we will administer the Euroqol EQ5D-5 L [51–53] at 6, 18, and 36 months [54]. The Canadian-specific EQ5D index score will be the primary quality of life measure [55].
3. Medication self-efficacy: we will administer the validated MASES questionnaire [56] at baseline and at study completion.
4. Necessity-concerns: we will administer the validated BMQ-specific questionnaire [57] at baseline and at study completion.

### Data management

Data entry for paper surveys will be done using iDataFax (DF/Net Research Inc I, Seattle, WA) with duplicate verification. All data will be stored in the study’s central data repository on secured University of Calgary servers within the Clinical Research Unit. Given the low-risk nature of our interventions, and since our outcomes will be assessed using administrative data (with a one-year lag to receipt of data), there will be no external data safety and monitoring board. This study is considered low risk since patients’ physicians remain ultimately responsible for managing patients’ medical treatments and any complications that may arise as part of their treatment.

### Statistical analysis

Primary outcome:

A Poisson model will test the main effects of the impact of the interventions on the rate of the primary outcome. This technique was chosen as individuals may experience multiple outcomes prior to the end of the study period, therefore we will account for both number of events and varying observation time within the Poisson model. The likelihood ratio test will test a negative binomial regression model within the Poisson model to examine for the presence of over dispersion. If present, negative binomial models will be used. The Vuong test will examine for excess zeros [58]. If present, zero-inflated models will be used.

Secondary outcomes:

Medication adherence is a binary variable and will be analyzed using log binomial regression (generalized linear models with a log link)—given the likely high prevalence of non-adherence. EQ5D index scores are continuous and will be analyzed using linear regression. Medication self-efficacy and concerns will be dichotomized and analyzed using logistic regression.

All analyses will be done using the “intention to treat” principle. Subgroup analyses will be based on underlying chronic disease and income status. Multiple imputations will be used to handle missing data.

### Cost-effectiveness analysis

Alongside this randomized trial, we will conduct a cost-utility analysis using the EQ5D index scores collected within the trial and administrative data sources to obtain costing information.

#### Background

Using the cohort of 170,000 Albertans noted above, cost of health care (physician, hospitalization, ambulatory care, and drug costs) was noted to exceed $12,000 per patient-year, with ~35 % of these costs related to components of the primary outcome (Table 1). We will assess whether reductions in the overall costs of care offset the cost of copayment elimination, or enhanced self-management education.

**Table 1:** Healthcare costs over a three-year period for a cohort of 170,000 Albertans noted as potentially eligible for this study using administrative health data

	No. of patients with an event within primary composite endpoint, or physician claim	Total events (including recurrent events) or physician claims	Percentage of patients with an event or physician claim	Average cost per patient over three years (includes those without any events)
Hospitalization events	47,201	110,773	48.7	$18,417
Physician claims	95,018	7,765,483	98.4	$5377
Ambulatory care / emergency department encounters	85,105	1,594,117	88.1	$5200
Prescription drug claims or costs	88,247	10,944,192	91.4	$5915
Sum of all above costs over 3 years				$36,332

#### Objective

The objective is to determine the cost per quality adjusted life year gained for the two study interventions in low-income seniors with chronic diseases.

#### Methods

We will calculate the total costs of care for patients randomized to each of the interventions. Using administrative data, the cost of physician visits, emergency department visits, chronic disease-related hospitalizations, outpatient interventions related to chronic diseases and medications will be calculated for each participant. Alberta Health uses CIHI case mix grouper methods and ambulatory case costing methods to estimate hospital and outpatient costs, respectively. Physician claims will be based on the amount paid by Alberta Health [59, 60]. If needed, extrapolation over a patient’s lifetime will be made using decision analysis [59, 60].

### Qualitative descriptive study

#### Overview

Qualitative research methods will be used to explore which intervention components did or did not work, why and in what contexts, by eliciting participants’ views and their reasons for non-adherence or adherence to self-management recommendations (including adherence to medications).

#### Sampling

A purposive sampling strategy will reflect: sex; type of chronic condition; income; and perception of a financial barrier. Five to ten participants will be selected from each of three active study arms (co-payment elimination only, self-management only, and both) across four groups of interest (those not on statins at baseline who started statins, those not on statins at baseline who remained off statins, those on statins at baseline with poor adherence but whose adherence improved, and those on statins at baseline with poor adherence and no improvement) resulting in a total of 12 groups. Sampling will continue to saturation, which we expect to occur at between 60-120 interviews.

#### Data collection

Telephone interviews will be conducted by a trained research assistant six months after enrolment to explore: general perceptions as to whether the intervention addressed individuals’ barriers; which aspects participants found helpful or not; why the intervention was successful at changing individual health behaviors or not; how the intervention affected individual’s quality of life (positively or negatively) and how the intervention could have been more impactful.

#### Data analysis

Qualitative descriptive/thematic analysis will be undertaken using qualitative data analysis and management software. Initial microcoding will break transcribed data into granular concepts, with subsequent coding to group concepts into themes. All analyses will be performed in duplicate by experienced qualitative data analysts.

#### Impact of qualitative study

In addition to gaining a better understanding of why the intervention(s) did or did not work, the results will be used to refine the self-management intervention that is provided in the remainder of the study, and will provide explanatory power for trial findings, especially if the interventions do not improve adherence and health outcomes.

## Discussion

Given the frequency of financial barriers to medication use in low-income Albertans, this study will test the impact of a value-based formulary which eliminates copayment for high-value preventive medication on outcomes and costs. Given the frequency of knowledge and motivation-related barriers, we will also assess the impact of a tailored self-management education and facilitated relay strategy which, if effective, could be rolled out in a cost-efficient manner to primary care clinics that have rosters of patients with chronic diseases. By comprehensively measuring the impact of the interventions on both health outcomes and costs, as well as the impact on reducing health inequities in this vulnerable population, our study will facilitate informed formulary policy decisions.

Many interventions that seek to change patient behavior have not been successful at improving adherence or improving outcomes [61, 62], so our team decided to take a different approach by incorporating input from both patients and commercial marketing/design firms in the intervention development phase. We believe that this novel academic, industry, and patient co-design approach will provide a framework for future initiatives.

By comprehensively measuring the impact of the interventions on both health outcomes and costs, as well as the impact on reducing health inequities in this vulnerable population, policy-maker partners will have all the information required to make informed formulary policy decisions.

We will present our findings at national and international conferences on health policy and drug financing. We aim to publish our findings in academic general medical journals. Authorship will be determined by the International Committee of Medical Journal Editors (ICMJE) criteria for authorship.

## Additional file

Additional file 1: Appendix 1. Medications included in copayment elimination intervention. **Appendix 2.** ICD10-CA definitions of the components of our primary clinical endpoint. **Appendix 3.** Alberta Kidney Disease Network Administrative Database. **Appendix 4.** Informed consent form. (DOCX 131 kb)
