# Supplementary material for: Assessing outcomes of enhanced chronic disease care through patient education and a value-based formulary study (ACCESS)—study protocol for a 2×2 factorial randomized trial
Source: Implement Sci. 2016 Sep 26;11:131. doi: 10.1186/s13012-016-0491-6 (PMC5037634; doi:10.1186/s13012-016-0491-6)
Supplement: Additional file 1: Appendix 1. — Medications included in copayment elimination intervention. Appendix 2. ICD10-CA definitions of the components of our primary clinical endpoint. Appendix 3. Alberta Kidney Disease Network Administrative Database. Appendix 4. Informed consent form. (DOCX 131 kb) [file 13012_2016_491_MOESM1_ESM.docx]

Appendix 1: Medications included in copayment elimination intervention

**Antiarrhythmic drugs**

Disopyramide *(Rythmodan*)

Procainamide *(Procan)*

Mexilentine *(Mexilentine)*

Flecainide (Tambocor)

Propafenone *(Propafenone/Rythmol)*

Amiodarone *(Amiodarone/Cordarone)*

Digoxin *(Toloxin)*

Sotalol (Sotacor, Betapace)

**Nitrates and nitrites**

Isosorbide Dinitrate (*Cedocard-SR)*

Isosorbide-5-Mononitrate *(Imdur)*

Nitroglycerin *(Nitrostat/Nitro/Nitrolingual/Nitro-dur/Trinipatch/Minitran/Nitrol)*

**Statins**

Atorvastatin *(Lipitor)*

Rosuvastatin *(Crestor)*

Simvastatin *(Zocor)*

Pravastatin *(Pravachol)*

Fluvastatin *(Lescol)*

Lovastatin *(Mevacor)*

**Non-statin Cholesterol lowering drugs**

Cholestyramine *(Olestyr)*

Colesevelam *(Lodalis)*

Colestipol *(Colestid)*

Bezafibrate *(Bezalip)*

Fenofibrate *(Feno-micro/Feno-Super/Lipidil Supra)*

Gemfibrozil (Lopid)

Ezetimibe *(Ezetrol)*

**Beta Blockers**

Acebutalol *(Sectral)*

Atenolol *(Tenormin/Tenoretic/Atenidone/*

*Atenolthalidone)*

Bisoprolol (Zebeta)

Carvedilol (Coreg, Coreg CR)

Labetalol *(Trandate)*

Metoprolol *(Lopresor)*

Propranolol (Inderal)

Nadolol *(Nadol)*

**ACE-inhibitors**

Benazepril *(Lotensin)*

Cilazepril *(Inhibace/Inhibace Plus)*

Enalapril *(Vasotec/ Vaseretic)*

Perindopril *(Coversyl/Coversyl Plus)*

Captopril *(Capoten)*

Fosinopril *(Monopril)*

Lisinopril *(Zestril/Prinivil/Zestoretic)*

Ramipril *(Altace)*

Quinapril *(Accupril/Accuretic)*

Trandolapril *(Mavik)*

**Angiotensin receptor blockers**

Candesartan *(Atacand/Atacand Plus)*

Eprosartan *(Teveten/Teveten Plus)*

Irbesartan *(Avapro / Avalide)*

Losartan *(Cozaar / Hyzaar)*

Telmisartan *(Micardis/Twynsta/ Micardis Plus)*

Valsartan *(Diovan)*

Olmesartan *(Olmetec/Olmetec Plus)*

**Calcium Channel blockers**

Nifedipine *(Adalat XL)*

Amlodipine *(Norvasc)*

Felodipine *(Plendil)*

Diltiazem *(Diltiaz/Cardizem / Tiazac)*

Verapamil *(Verap/Isoptin SR)*

**Diuretics**

Hydrochlorothiazide *(Hydrazide/Hydro)*

Furosemide *(Lasix)*

Spironolactone *(Aldactone / Aldactazide)*

Indapamide *(Lozide)*

Metolazone *(Zaroxolyn)*

Chlorthalidone (Thalitone)

Amiloride *(Midamor/ Novamilor /Amilizide)*

Triamterene *(Triazide*)

Ethacrynic Acid *(Edecrin)*

**Other Blood Pressure Medications**

Clonidone *(Catapres/Clonidine)*

Methyldopa (Aldomet)

Hydralazine (Apresoline)

Minoxidil *(Loniten)*

Doxazosin *(Cardura)*

Prazosin *(Prazo)*

Terazosin *(Hytrin)*

**Anticoagulants**

Warfarin *(Coumadin)*

Rivaroxaban *(Xarelto)*

Dabigatran *(Pradaxa)*

Apixaban *(Eliquis)*

Dalteparin *(Fragmin)*

Tinzaparin *(Innohep)*

Enoxaparin *(Lovenox)*

Heparin *(Heparin Leo)*

Nadroparin *(Fraxiparine)*

Fondaparinux *(Arixtra)*

Danaparoid *(Orgaran)*

**Anti-diabetes Medications**

Metformin *(Glucophage)*

Glipizide (Glucotrol)

Gliclazide *(Diamicron)*

Glyburide *(Diabeta)*

Glibenclamide (Euglucon)

Acarbose *(Glucobay)*

Repaglinide *(Gluconorm)*

Linagliptin *(Trajenta/Jentadueto)*

Saxagliptin *(Onglyza)*

Sitagliptin *(Januvia/Janumet)*

Pioglitazone *(Actos)*

Rosiglitazone *(Avandia/Avandamet)*

**Anti-platelet agents**

Clopidogrel *(Plavix)*

ASA*-Dipyridamole (Aggrenox)*

Ticagrelor *(Brilinta)*

**Insulin**

Insulin Aspart (Novorapid)

Insulin Detemir (Levemir)

Insulin Glargine *(Lantus)*

Insulin Glulisine *(Apidra)*

Insulin R *(Novolin/Humulin)*

Insulin Lispro *(Humalog)*

Insulin Humulin 30/70

Insulin Humulin N

Insulin Humulin R

Insulin Novolin NPH

Insulin Novolin Toronto

Insulin Novolin Mix (30/70, 40/60, 50/50)

**Smoking cessation Aids**

Varenicline *(Champix)*

# Appendix 2 – ICD10-CA definitions of the components of our Primary clinical Endpoint

| **Outcome** | **Description** | | | **Classification Source** | **Codes included** | | **Exclusions** | **Source** |
| --- | --- | --- | --- | --- | --- | --- | --- | --- |
| **Myocardial Infarction** |  | | |  |  | |  |  |
|  | Acute myocardial infarction  Subsequent myocardial infarction | | | ICD-10 | I21.X  I22.X | | None | Quan et al. [[47](#_ENREF_47)], Austin et al. [[43](#_ENREF_43)] |
| **Stroke** | Central retina artery occlusion  Cerebral infarction  Stroke, not specified as hemorrhage or infarction  Intracerebral hemorrhage  Subarachnoid hemorrhage  Transient cerebral ischemic attacks | | | ICD-10 | H34.1  I63.X  I64.X  I61.X  I60.X  G45.X | | None | Kokotailo and Hill [[44](#_ENREF_44)] |
| **Coronary Revascularization** | Coronary Angioplasty  Coronary endarterectomy/excision  Coronary local pharmacotherapy  Coronary Artery Bypass | | | Canadian Classification of Health Interventions (CCI) | 1.IJ.50  1.IJ.57  1.IL.35  1.IJ.76 | |  | CIHI |
| **Death** | Death (all-cause) | | | Vital Statistics |  | |  |  |
| **Ambulatory Care-Sensitive Hospitalization for**  **Diabetes** | | Type 1 DM with coma |  | | E10.0^^ | None | | CIHI |
|  |  | Type 1 DM with acidosis |  | | E10.1^^ |  | |  |
|  |  | Type 1 DM with hypoglycaemia |  | | E10.63 |  | |  |
|  |  | Type 1 DM without (mention of) complication |  | | E10.9^^ |  | |  |
|  |  | Type 2 DM with coma |  | | E11.0^^ |  | |  |
|  |  | Type 2 DM with acidosis |  | | E11.1^^ |  | |  |
|  |  | Type 2 DM with hypoglycaemia | ICD-10 | | E11.63 |  | |  |
|  |  | Type 2 DM without (mention of) complications |  | | E11.9^^ |  | |  |
|  |  | Other specified DM with coma |  | | E13.0^^ |  | |  |
|  |  | Other specified DM with acidosis |  | | E13.1^^ |  | |  |
|  |  | Other specified DM with hypoglycaemia |  | | E13.63 |  | |  |
|  |  | Other specified DM without (mention of) complication |  | | E13.9^^ |  | |  |
|  |  | Unspecified DM with coma |  | | E14.0^^ |  | |  |
|  |  | Unspecified DM with acidosis |  | | E14.1^^ |  | |  |
|  |  | Unspecified DM with hypoglycaemia |  | | E14.63 |  | |  |
|  |  | Unspecified DM without (mention of) complication |  | | E14.9^^ |  | |  |
| **Ambulatory Care-Sensitive Hospitalization for**  **Chronic Kidney Disease.** | | Type 2 diabetes mellitus with ketoacidosis  Type 1 diabetes mellitus with ketoacidosis  Type 2 diabetes mellitus with ketoacidosis with lactic acidosis  Type 1 diabetes mellitus with ketoacidosis with lactic acidosis |  | | E11.10  E10.10  E11.12  E10.12 | None | | Gao et al.[[38](#_ENREF_38)] |
|  |  | Type 2 diabetes mellitus with coma |  | | E11.00 |  | |  |
|  |  | Type 1 diabetes mellitus with coma | ICD-10 | | E10.00 |  | |  |
|  |  | Hyperosmolality and hypernatraemia |  | | E87.0 |  | |  |
|  |  | Hyperkalaemia |  | | E87.5 |  | |  |
|  |  | Fluid overload |  | | E87.7 |  | |  |
|  |  | Malignant hypertension |  | | I10.1 |  | |  |
|  |  | Heart failure |  | | I50.x |  | |  |
|  |  | Hypertensive heart disease |  | | I11.0 |  | |  |
|  |  | Hypertensive heart and renal disease |  | | I13.0 |  | |  |
|  |  | Hypertensive heart and renal disease |  | | I13.2 |  | |  |
|  | | Ischaemic cardiomyopathy |  | | I25.5 |  | |  |
|  |  | Dilated cardiomyopathy |  | | I42.0 |  | |  |
|  |  | Other restrictive cardiomyopathy |  | | I42.5 |  | |  |
|  |  | Alcoholic cardiomyopathy |  | | I42.6 |  | |  |
|  |  | Cardiomyopathy due to drugs and other external agents |  | | I42.7 |  | |  |
|  |  | Other cardiomyopathies |  | | I42.8 |  | |  |
|  |  | Cardiomyopathy, unspecified |  | | I42.9 |  | |  |
| **Ambulatory Care-Sensitive Hospitalization for**  **Hypertension** | | Benign hypertension |  | | I10.0 | See below* | | CIHI |
|  |  | Malignant hypertension |  | | I10.1 |  | |  |
|  |  | Hypertensive heart disease |  | | I11 |  | |  |
| **Ambulatory Care-Sensitive Hospitalization for**  **Heart Failure** | | Rheumatic heart disease, unspecified |  | | I109.9 | None | |  |
|  |  | Hypertensive Heart Disease |  | | I11.0 |  | | CIHI |
|  |  | Hypertensive Heart and Renal Disease and stage 1 through stage 4 chronic kidney disease, or unspecified chronic kidney disease |  | | I13.0 |  | |  |
|  |  | Hypertensive heart and chronic kidney disease without heart failure, with stage 1 through stage 4 chronic kidney disease, or unspecified chronic kidney disease |  | | I13.10 |  | |  |
|  |  | Hypertensive Heart And Renal Disease and with stage 5 chronic kidney disease, or end stage renal disease |  | | I13.2 |  | |  |
| **Ambulatory Care-Sensitive Hospitalization for**  **Coronary artery disease** | | Angina pectoris |  | | I20 | See below* | |  |
|  |  | Other current complications following acute myocardial infarction |  | | I23.82 |  | | CIHI |
|  |  | Acute coronary thrombosis not resulting in myocardial infarction |  | | I24.0 |  | |  |
|  |  | Other forms of acute ischemic heart disease |  | | I24.8 |  | |  |
|  |  | Acute ischemic heart disease, unspecified |  | | I24.9 |  | |  |

* Excludes hospitalizations with one of the following cardiac procedure codes: 
CCP: 47^^, 480^–483^, 489.1, 489.9, 492^–495^, 497^, 498^
ICD-9-CM: 336, 35^^, 36^^, 373^, 375^, 377^, 378^, 379.4–379.8 
CCI: 1HA58, 1HA80, 1HA87, 1HB53, 1HB54, 1HB55, 1HB87, 1HD53, 1HD54, 1HD55, 1HH59, 1HH71, 1HJ76, 1HJ82, 1HM57, 1HM78, 1HM80, 1HN71, 1HN80, 1HN87, 1HP76, 1HP78, 1HP80, 1HP82, 1HP83, 1HP87, 1HR71, 1HR80, 1HR84, 1HR87, 1HS80, 1HS90, 1HT80, 1HT89, 1HT90, 1HU80, 1HU90, 1HV80, 1HV90, 1HW78, 1HW79, 1HX71, 1HX78, 1HX79, 1HX80, 1HX83, 1HX86, 1HX87, 1HY85, 1HZ53 rubric (except 1HZ53LAKP), 1HZ55 rubric (except 1HZ55LAKP), 1HZ56, 1HZ57, 1HZ59, 1HZ80, 1HZ85, 1HZ87, 1IF83, 1IJ50, 1IJ55, 1IJ57, 1IJ76, 1IJ86, 1IJ80, 1IK57, 1IK80, 1IK87, 1IN84, 1LA84, 1LC84, 1LD84, 1YY54LANJ

**Appendix 3 – Alberta Kidney Disease Network Administrative Database**

**Appendix 4: Informed Consent Form**

**Department of Medicine**

**Informed Consent Form**

**A**ssessing outcomes of enhanced **C**hronic disease Care through patient **E**ducation and a value-ba**S**ed formulary **S**tudy (**ACCESS**)

**Study Coordinating Group:**

Interdisciplinary Chronic Disease Collaboration (ICDC)

Department of Medicine, Division of Nephrology,

University of Calgary

**Investigators:**

Dr. Braden Manns, Principal Investigator, University of Calgary

Dr. David Campbell, Co-Investigator, University of Calgary

Dr. Marcello Tonelli, Co-Investigator, University of Calgary

Dr. Brenda Hemmelgarn, Co-Investigator, University of Calgary

**Study Coordinating Group Location:**

G230 Health Sciences Centre

3330 Hospital Drive, N.W.

Calgary, Alberta T2N 1N4

**Contact Information:** (403) 210-6609 or toll free at 1-844-310-0585

Thank you for your interest in the ACCESS trial. In addition to the details provided to you over the phone when you consented to provide us with some information, the purpose of this consent form is to provide you with a basic idea of the objectives of this research study and what your participation will entail. Take the time to read this information carefully. If you would like more detail about anything mentioned here, or information not included here, please contact

| **BACKGROUND** |
| --- |

| **WHAT IS THE PURPOSE OF THE STUDY?** |
| --- |

Many people are living with chronic health conditions, like diabetes, high blood pressure or heart disease. Chronic conditions often occur together and can lead to heart attacks and strokes, which are leading causes of death and disability. Although effective therapies for these conditions, including medications and lifestyle changes, are available, many people do not receive these treatments.

The purpose of this study is to look at ways to improve health for people with chronic health conditions like the ones you have. In this three year study, we are testing the impact of two interventions to which you will be randomly assigned:

**1) Preventive medications free of charge:** receiving preventive medications for diabetes, high blood pressure, or heart disease without copayment through your regular Alberta Blue Cross Coverage for Seniors Program (please see the attached medication guide which provides more information on the preventive medications that would be provided free of charge) and

**2) Personalized education program to help you manage your chronic conditions:** receiving a comprehensive patient education program tailored to your needs based on the information you provide to us at the start of the study.

Since there are two interventions, you will be randomly selected to be in one of following four study groups:

(1) both **preventive medications free of charge** AND free enrollment in a new **personalized education program to help you manage your chronic conditions**,

(2) **preventive medications free of charge** AND access to educational information about your chronic conditions;

(3) continue with your usual Blue Cross drug insurance coverage and free enrollment in a new **personalized education program to help you manage your chronic conditions** ;

(4) continue with your usual Blue Cross drug insurance coverage and access to educational information about your chronic conditions.

If you are randomly assigned to receive **preventive medications free of charge** , you will continue to receive drug coverage through Alberta Blue Cross Coverage for Seniors Program throughout this study, and at the end of the study (3 years after enrolment), your drug coverage will return to your current plan (in other words, **you will start paying your copayment again**).If you are randomized to the **personalized education program**, your personalized education will also end at the end of study (3 years after enrolment).

If you are randomized to the **personalized education program**, you will have access to personalized education by mail or electronically depending on your preference. If you choose to be contacted electronically, as part of the educational platform, we may send you emails containing information about the medications you take and your health related behaviors.

| **WHAT DOES MY PARTICIPATION INVOLVE?** |
| --- |

When you enter the study you will be asked to complete a short survey containing questions about your chronic conditions, your medications, your comfort with the health system and any barriers that you may experience when receiving care for your chronic conditions. You will complete these surveys either electronically through a secure website or on paper, depending on your preference.

Questionnaires similar to the one completed at the start of the study will also be administered to you again at 6 months, 18 months and when you reach the end of the study at three years. These questionnaires will be shorter and will take approximately 10 minutes to complete. Based on your preference, they can be completed online through the secure website, or mailed out to you to complete and return. If you selected the mail option; the cost of the postage will be covered by the study.

It is important to keep in mind that you do not have to share any information that you are not comfortable sharing and you are free at any time to refuse to answer any of the questions.

In addition to the questionnaires, over the course of the study, we will also be collecting necessary information from your personal health records stored with Alberta Health, Alberta Blue Cross and Alberta Health Services. This information is needed in order to help us determine what medications you are taking, the costs of the medications you are taking, and whether you are hospitalized or develop other medical problems. We will also collect laboratory test information, as well as information on your physician visits. This information will be used to help us determine the overall costs of the healthcare and related health care services provided to you.

Finally, we may call you at a later date to ask if you are willing to participate in a brief telephone interview. You are free to decline to participate in this aspect of the study.

| **YOUR PARTICIPATION** |
| --- |

There will be no payment to you for participation in this study and you will not be charged for taking part in this study.

Taking part in this research study is completely voluntary. You can change your mind and withdraw (drop out) at any time without jeopardizing your health care. If you decide to withdraw, no further information will be collected about you. Any information previously collected will be kept for research purposes unless you personally request the withdrawal of this data.

| **WHAT ARE THE RISKS?** |
| --- |

No physical risks, harms or discomfort associated with this type of research have been identified. However, it is not possible to know all of the risks that may happen in a study, but the researchers have taken all reasonable safeguards to minimize any known risks to study participants.

In the event that you suffer injury as a result of participating in this research, no compensation will be provided to you by the University of Calgary, the Alberta Health Services, Alberta Health, Alberta Blue Cross or the Researchers. You still have all your legal rights. Nothing said in this consent form alters your right to seek damages.

| **WHAT ARE THE BENEFITS?** |
| --- |

There may or may not be a direct benefit to you from participating in this study. You are in the study because you have been identified as having a chronic condition that may be improved during the study but there is no guarantee that this research will help you. The information we get from this study may help us to provide better treatments and programs in the future for patients with chronic conditions like yours.

| **PRIVACY OF YOUR RECORDS** |
| --- |

The data collected for this study will be kept strictly confidential. Your research information may be used and disclosed for the purposes of this study only. Specifically, if you are randomized to the copayment elimination arm, we would need to disclose some identifying information including your name, date of birth, address and personal health number to Alberta Blue Cross to change your pharmaceutical coverage. We will not use or disclose the information collected in this study for another research purpose without your written permission.

Your name will not appear in the data collected only an assigned code number and your initials and date of birth will be used. Your identity will be kept confidential at all times and no information that could identify you will be put in any report published from the study. Your data will be stored on a secure firewalled servers on the University of Calgary campus. In assisting us to mail items to you, your name and mailing address may be shared with Canada Post mailing services employed by the University of Calgary.

By giving us your written consent to participate, you give your permission to the investigators (study researchers) to access any personally identifiable health information, which is under the custody of other health care professionals as deemed necessary for the conduct of this research. This includes access to health information in the past and might include data held in provincial sources by Alberta Health, Alberta Health Services and Alberta Blue Cross. You have also given your permission for us to periodically contact you by telephone, electronic means such as through your personal email address and / or by postal mail, whichever method you have indicated as preferred.

The University of Calgary Conjoint Health Research Ethics Board has approved this research study. The University requires us to keep data from the study for five years after the study is completed. All data and study information will be kept in a locked research office and stored on secure servers that are approved to house patient identifiable information.

| **WHAT IF I HAVE QUESTIONS?** |
| --- |

If you would like more information on the study, or if you have further questions concerning matters related to this research, please contact the Study Coordinating Group at (403) 210-6609 or toll free at 1-844-310-0585.

If you have any questions concerning your rights as a study participant in this research, or research in general, please contact the Chair of the Conjoint Health Research Ethics Board, University of Calgary at (403) 220-7990, or the University of Alberta Health Research Ethics Board at (780) 492-2615

| **AGREEMENT TO PARTICIPATE** |
| --- |

Your decision to complete and return this consent form and the survey questionnaire will be interpreted as an indication of your agreement to participate. In no way does this waive your legal rights nor release the investigators, or involved institutions from their legal and professional responsibilities. You are free to withdraw from the study at any time without jeopardizing your health care.

If you have further questions concerning matters related to this research, please contact:

Dr. Braden Manns (403) 944-2595

If you have any questions concerning your rights as a possible participant in this research, or research in general, please contact the Chair of the Conjoint Health Research Ethics Board, University of Calgary at (403) 220-7990, or the University of Alberta Health Research Ethics Board at (780) 492-2615.

| **INFORMED CONSENT FORM** |
| --- |

**By signing below, I confirm that:**

I have read and understand the information sheet for the above research study.

I know why I have been asked to disclose my individually identifying information and I am aware of the risks and benefits of participating in the study.

The study has been explained to me and I have had the opportunity to consider the information, ask questions, have had my questions answered to my satisfaction, and have had time to decide whether to participate. I know who to contact if I have any questions.

I understand that my participation is voluntary and that I am free to withdraw at any time without my medical care or legal rights being affected.

I agree that my coded personal data can be archived at the end of the study.

I understand I will receive a signed original of this Patient Information and Consent Form for my records.

I agree to take part in this research study

| Participant’s Name (Please Print) |  | Signature  ____________________________  Date |
| --- | --- | --- |
|  |  |  |

The University of Calgary Conjoint Health Research Ethics Board has approved this research study.

Please print and keep a copy of this consent form for you records and reference.
